# Living in a large family and low daily water consumption substantially expose for human scabies in rural Ethiopia: a matched analysis

**DOI:** 10.1186/s41043-023-00471-6

**Published:** 2023-11-28

**Authors:** Agernesh Ayele, Metadel Adane, Balew Adane, Gete Berihun, Mesfin Gebrehiwot, Lebasie Woretaw, Leykun Berhanu, Getu Atanaw, Hailemariam Feleke, Mekonnen Moges, Eniyew Tegegne, Jember Azanaw, Asmamaw Malede

**Affiliations:** 1 Department of Environmental Health, Wollo University, Dessie, Ethiopia; 2 Department of Environmental Health, Debre Markos University, Debre Markos, Ethiopia; 3 Department of Obstetrics and Gynecology, University of Gondar, Gondar, Ethiopia; 4 Department of Environmental and Occupational Health and Safety, University of Gondar, Gondar, Ethiopia

**Keywords:** Amhara Region, Matched analysis, Rural community, Scabies cases

## Abstract

**Background:**

Scabies has been added to the neglected tropical diseases portfolio for large-scale disease control action since 2017 and is part of the WHO roadmap for NTDs 2021–2030, targeted at ending the neglect to achieve the sustainable development goals. Previous studies have not fitted matched analysis to identify predictors of scabies infestation in Ethiopia. Information is also scarce about predictors of scabies infestation in this area. Therefore, this study aimed to identify predictors of scabies infestation in rural *Aneded* District, northwest Ethiopia.

**Methods:**

A community-based matched case–control study involving 183 cases and 549 controls was undertaken from March 1 to May 31, 2021, in rural *Aneded* District. A two-stage sampling technique with a house-to-house census for the screening of scabies cases was employed. A structured questionnaire with questions on sociodemographics, behavior, water supply, sanitation, and hygiene, and delivery of scabies-specific interventions was used. Pretesting, training of data collectors and supervisors, and supervision were applied to keep the data quality. A multivariable conditional logistic regression model was fitted to identify predictors of scabies.

**Results:**

Unmarried individuals or those in separated families (adjusted matched odds ratio (AmOR = 2.71; 95% CI 1.30–5.65); those unable to read and write or in illiterate families (AmOR = 5.10; 95% CI 1.81–14.36); those in large families (AmOR = 6.67; 95% CI 2.83–15.73); households that had longer travel times for water collection (AmOR = 2.27; 95% CI 1.08–4.76); those that had low daily water consumption (AmOR = 6.69; 95% CI 2.91–15.37); households that disposed of solid wastes in open fields (AmOR = 5.60; 95% CI 2.53–12.40); and households that did not receive scabies-specific interventions (AmOR = 2.98; 95% CI 1.39–6.39) had increased odds of scabies.

**Conclusions:**

Being unmarried, illiteracy, large family, long travel time for water collection, low daily water consumption, open dumping of solid wastes, and inaccessibility of scabies-specific interventions are predictors of scabies. This information is instrumental for redesigning improved scabies-specific interventions that consider educational status, marital status, family size, water collection time, daily water consumption, solid waste disposal, and equity and optimization in delivering existing interventions in rural Ethiopia.

## Background

Scabies is an underrecognized, unacceptably highly prevalent, transmittable ecto-parasitic skin disease of humans and is responsible for considerable illnesses and a few deaths. It generally occurs in all countries; nonetheless, most cases occur in low-income and middle-income countries. Globally, it causes 71 disability-adjusted life-years (DALYs) per 100,000 people and accounts for 0.21% of DALYs from all conditions. This burden is equivalent to that caused by *Haemophilus influenzae* type b, meningitis and acute lymphoid leukaemia. The disease burden is greater in tropical regions, especially in children, youths, and elderly people [1]. Globally, it infests 100 to 200 million people at any time, with a prevalence ranging from 0.2 to 71% [2].

Scabies is a public health issue in Ethiopia, particularly in disadvantaged communities. A systematic review and meta-analysis study indicated a 14.5% pooled prevalence of scabies infestation in Ethiopia, which was highest in the Amhara Region (19.6%) and highest among young people [3]. In the Amhara region, the prevalence in 68 districts varied from 2 to 67%, with a median prevalence of 33.5%, and nearly half of the cases were school-aged children [4].

The causative agent for scabies is an obligate microscopic female mite named *Sarcoptes scabiei* var *hominis*. Scabies causes severe itching and stigmatizing skin lesions. Furthermore, it leads to impetigo, severe bacterial superinfections, and postinfectious complications, including septicaemia, possibly renal disease, and rheumatic heart disease [5–7].

Scabies is a critical public health concern where there is societal breakdown, congestion, and poor domestic and personal hygiene. It is typically transmitted by prolonged skin contact with a mite-infested person or seldom by sharing infested personal entities, and zoonotic transmission does not occur [8–11]. Scabies transmission increases in high population density circumstances; a proxy of high endemicity in poorest communities resulted from crowded housing and was observed at outbreaks in institutions, such as prisons, aged care facilities, schools, hospitals and refugee camps [12, 13].

In prior studies, illiteracy [9, 10, 14–17], large family [3], low socioeconomic status [8, 9, 17, 18], overcrowding [9, 16, 18, 19], inadequate hygiene [8, 14, 16], sharing fomites [3, 8–10, 15], absence of healthcare service [20, 21], environmental sanitation [8, 11, 22–24], and dementia [13] were identified as underlying factors for scabies infestation.

In 2017, the WHO Department of Neglected tropical disease (NTD) Control formally added scabies to the NTD portfolio for large-scale disease control action through core strategies: preventive chemotherapy using mass drug administration (MDA) and/or innovative and intensified case management [25]. The disease is being adopted into integrated programs of MDA for NTDs through combination MDA to control multiple NTDs simultaneously in communities [26, 27]. Likewise, it has recently been included as part of the WHO roadmap for NTDs 2021–2030, targeted at ending the neglect to achieve the Sustainable Development Goals [28]. In Ethiopia, scabies has been included as a reportable disease in drought-prone areas since 2015. However, it is becoming a public health problem in wide geographic areas and populations [4, 29].

Scabies infestations have received broad neglect across multiple research domains due to their low death outcome [25, 30]. To date, studies on scabies infestation in Ethiopia have not used conditional logistic regression model to identify independent predictors. Additionally, there is no information about the predictors of scabies infestation in rural communities of the study area. Thus, this study aimed to detect the association of sociodemographics, water supply and sanitation, and scabies-specific interventions with scabies infestations in rural households (HHs) of Ethiopia. The findings of this study may help guide national NTD intervention programs and health authorities to redesign improved cost-effective and comprehensive integrated strategies for scabies prevention and control combined with other NTDs in Ethiopia.

## Methods

### Study area description and period

The study was carried out in nineteen rural *kebeles* (the lowest administrative units in Ethiopia) of *Aneded* District, northwest Ethiopia, from March 1 to May 31, 2021. The district has three urban *kebeles*. In 2019/2020, the district had a projected population of 110,183 (53,989 males and 56,194 females) residents [31] and 4731 HHs. It had 4 health centers and 20 health posts. The district has two temperature-based agro-ecological zones: midlands (most of the *kebeles*) and lowlands. The district is largely characterized by good socioeconomic status, adequate and improved water supply (mainly public standpipes), good sanitation coverage, and moderate personal hygiene practice.

### Study design and eligibility criteria

A community-based matched case–control study was used. Individual matching by sex and category matching by age (below 15, 15–49, and above 49 years) for cases and controls were employed. All individuals aged 1 year and above who were permanent residents (had lived for at least 6 months) in the rural *kebeles* of the district were included. Individuals who had other dermal illnesses or severe diseases except scabies were excluded from this study.

### Sample size calculation

The sample size was estimated using a matched case–control study design calculation formula (32) by assuming a 95% confidence level, 90% power to observe exposure variability, an odds ratio (OR) of 1.75, a 1:3 case–control ratio, and considering that 36.3% of the control HHs had water accessibility for personal hygiene [33, 34]. In addition, a 10% nonresponse rate was considered for both the estimated sample size and the nonrespondent sample and computed using the formula (final sample size = $n/1{-}10\%$). As a result, a matched sample size of 190 pairs (190 cases and 570 controls) could be enrolled in the study.

### Study population and sampling technique

A two-stage sampling technique was used to select the study subjects. In the first stage, five rural *kebeles*, including *Enaskay*, *Mislewash*, *Wonganefasam*, *Daget*, and *Yewush* (30% of the total rural *kebeles*), were included using the lottery method from the nineteen rural *kebeles* of the district. In the second stage, a house-to-house census/visit was performed to screen individuals having scabies infestation in the five *kebeles*. In each HH, all members were screened for scabies status. Then, sampling frames were created during house-to-house visit in each *kebele*. Finally, a proportional sample of matched controls (matched by sex and age) was taken from each *kebele* on the basis of the number of scabies cases. For selection of matched controls, simple random sampling was employed. Controls were selected from the same *kebele* where the cases were lived. When there was more than one scabies case or control in an HH, only one was selected randomly. Study subjects (scabies cases and controls) were included in the study based on the WHO case definition for scabies [35, 36].

### Data collection and quality assurance

A consensus diagnostic criterion using the Delphi method was used to confirm scabies infestation in the study population. The Delphi method is the standardized and accepted diagnostic method for scabies [36]. Cases were individuals who had clinical signs and symptoms of scabies, with at least scabies burrows, typical lesions affecting male genitalia, or typical lesions in a typical distribution and two history features (Level B) [35, 36]. Controls were individuals who had no clinical signs or symptoms of scabies. Experienced public health professionals who were certified by the Ethiopian Federal Ministry of Health confirmed whether the individual had scabies using the Delphi method. One-day training was given for the public health professionals for the diagnosis of scabies.

A pretested structured questionnaire was used to collect sociodemographic, socioeconomic, behavioral, water supply, sanitation, hygiene/WaSH related, and health service coverage characteristics of the study participants. Face-to-face interviews and/or direct observations were used to gather data.

Questionnaire-based data were collected by Environmental Health professionals with a Bachelor of Science degree. Three-day training was given by the lead researcher for data collectors about house-to-house data collection. The questionnaire was pretested on 5% of the sample size in a rural *kebele* in *Gozamin* district. Strict follow-up was applied to optimize the quality of data through trained supervisors. If any scabies case or control was not present at the first visit in house-to-house data collection, the HH was revisited for the second time. House-to-house data collectors were blinded to the scabies status of the study subjects. The questionnaire was first developed in English and translated into the Amharic language, and only the open responses from the study subjects were translated to English by independent language professionals.

### Variable measurements

Variables included in the study were defined as follows: 1) scabies infestation is the presence of itching with typical lesions on hands, interdigital, and/or genitalia and/or itching and close contact with an individual who had itching or typical lesions in a typical distribution [36]; 2) when an individual did not take a shower at least once in a week, it was defined as infrequent bathing; 3) when a person did not wash his/her clothes at least once in a week, the variable was measured as infrequent cloth washing; 4) when a person did not change his/her clothes at least once in a week, infrequent cloth changing was used as a definition; 5) daily water consumption of ≥ 20 L/capita/day (L/C/D) was considered as adequate water quantity; 6) disposal of HH solid wastes by using sacks, disposal pit, or composting in the compound and liquid wastes by using septic tank or soak pit in the compound was defined as proper waste management; and 7) receiving scabies-specific interventions was defined as receiving health interventions, including mass therapy, health education, training, and ongoing surveillance.

The HH wealth index for rural Ethiopia was constructed from several binary and ordinal variables adapted from the literature [37] and the Ethiopian Demographic and Health Survey [38]. Variables, including longer-run and shorter-run HH assets, latrine availability and type, housing characteristics, farmland size, ownership and number of domestic animals, and beekeeping, were used to compose the wealth index. Variables had values that ranged from 0 for the lowest to 1 for the highest value.

### Data analysis

Data entry, coding, cleaning and verification were performed in *EpiData Version 3.1* software (EpiData Association, Odense, Denmark). However, data analysis was performed in SPSS statistical software version 24 (IBM SPSS Statistics for Windows; NY, USA) and STATA version 14.0 (Statistical Software: College Station, TX 77845, USA). The median with IQR (interquartile range) was computed for continuous data. Frequencies and proportions were calculated for categorical data.

Principal component analysis (PCA) was run to describe HH wealth [37]. Principal component 1 was considered to rate the HHs into three categories. Cut-off values were determined for the three ranks. The original variables were standardized. Standardized variables were multiplied by principal component 1 and resulted in third variables. Ultimately, a composite variable, the HH wealth score, was derived by summing the third variables. By applying the cut-off values of principal component 1, a variable, the HH wealth index with three categories, was defined. The variable was categorized into tertiles, with the lower tertile indicating lower wealth.

Bivariable and multivariable conditional logistic regression models were fitted to detect predictors of scabies infestation. A bivariable matched analysis was performed, and variables having *P* < 0.25 along with variables having scientific meaning were included in the multivariable matched analysis. Adjusted matched odds ratios (AmORs) with 95% confidence intervals (CIs) and *P*-values were reported for the multivariable matched analysis. Variables at *P* < 0.05 were declared statistically significant predictors of scabies infestation.

## Results

### Sociodemographic characteristics

A total of 732 study participants (183 scabies cases and 549 controls) participated in the study, yielding a response rate of 96.3%. Seven matched pairs were not enrolled in the study because the HHs refused to be included. The matching proportion of age through scabies cases and controls was as follows: 308 (42.1%) for participants under 15 years of age, 224 (30.6%) for participants 15–49 years of age and 200 (27.3%) for participants above 49 years of age. The median (IQR) age was 17 (11–50) years for the scabies patients and 17 (11–52) years for the controls. Out of 183 patients with scabies, 82 (44.8%) were males and 101 (55.2%) were females; among 549 controls, 246 (44.8%) were males and 303 (55.2%) were females (Table 1).

**Table 1:** Sociodemographic characteristics of study participants in rural areas of *Aneded District*, northwest Ethiopia, March–May 2021

Variables	Scabies cases (*n* = 183) *n* (%)	Controls (*n* = 549) *n* (%)	Total *n* (%)
*Religion*^a^			
Christian	181 (98.9)	540 (98.4)	721 (98.5)
Muslim	2 (1.1)	9 (1.6)	11 (1.5)
*Marital status a*			
Married	104 (56.8)	413 (75.2)	517 (70.6)
Unmarried	79 (43.2)	136 (24.8)	215 (29.4)
*Educational level*^a^			
Able to read & write^b^	26 (14.2)	209 (38.1)	235 (32.1)
Unable to read & write	157 (85.8)	340 (61.9)	497 (67.9)
*Occupation a*			
Farmer & merchant	166 (90.7)	521 (94.9)	687 (93.8)
Daily laborer	17 (9.3)	28 (5.1)	45 (6.2)
*Family size*			
1–4 individuals	36 (19.6)	375 (68.3)	411 (56.2)
≥ 5 individuals	147 (80.4)	174 (31.7)	321 (43.8)
*HH wealth index*			
Rich	89 (48.6)	236 (43.0)	325 (44.4)
Medium	4 (2.2)	8 (1.4)	12 (1.6)
Poor	90 (49.2)	305 (55.6)	395 (54.0)

^a^For study participants under 18 years; religion, marital status, educational status & occupation of the HH head were taken

^b^Formal and nonformal education

### Water supply, sanitation and hygiene characteristics

More than half of the scabies case-HHs, 103 (56.3%), obtained water from public standpipes and 101 (55.2%) took more than 30 min to go to the water source and return to home. One hundred five (57.4%) of the scabies patients consumed less than 20 L of water in a day for daily activities. Nearly two-thirds of the scabies case-HHs, 116 (63.4%), disposed of the generated solid wastes in the open field (Table 2).

**Table 2:** Water supply, sanitation and hygiene characteristics of HHs and study participants in rural areas of *Aneded District*, northwest Ethiopia, March–May 2021

Variables	Scabies cases (*n* = 183) *n* (%)	Controls (*n* = 549) *n* (%)	Total *n* (%)
*Water source type*			
Public standpipe	103 (56.3)	463 (84.3)	566 (77.3)
Unprotected spring & well	80 (43.7)	86 (15.7)	166 (22.7)
*Water collection time*^a^			
1–30 min	82 (44.8)	462 (84.2)	544 (74.3)
> 30 min	101 (55.2)	87 (15.8)	188 (25.7)
*Daily water consumption (average)*			
≥ 20 L/C/D	78 (42.6)	458 (83.4)	536 (73.2)
< 20 L/C/D	105 (57.4)	91 (16.6)	196 (26.8)
*Sanitation facility type*			
Pit latrine with cover slab	136 (74.3)	507 (92.4)	643 (87.8)
Pit latrine without cover slab	47 (25.7)	42 (7.6)	89 (12.2)
*Solid waste disposal*			
Proper^b^	67 (36.6)	403 (73.4)	470 (64.2)
Open	116 (63.4)	146 (26.6)	262 (35.8)
*Liquid waste disposal*			
Using soak pit	4 (2.2)	11 (2.0)	15 (2.1)
Open discharging	179 (97.8)	538 (98.0)	717 (97.9)
*Bathing frequency*			
Weekly & below	9 (4.9)	53 (9.6)	62 (8.5)
Every other week & above	174 (95.1)	496 (90.4)	670 (91.5)
*Cloth washing frequency*			
Weekly & below	6 (3.3)	57 (10.4)	63 (8.6)
Every other week & above	177 (96.7)	492 (89.6)	669 (91.4)
*Using soap for bathing*			
Yes	148 (80.9)	529 (96.4)	677 (92.5)
No	35 (19.1)	20 (3.6)	55 (7.5)
*Cloth changing frequency*			
Weekly & below	171 (93.4)	523 (95.3)	694 (94.8)
Every other week & above	12 (6.6)	26 (4.7)	38 (5.2)
*History with a scabies case*^c^			
No	108 (59.0)	527 (96.0)	635 (86.8)
Yes	75 (41.0)	22 (4.0)	97 (13.2)
*Cloth mixing history with scabies case*			
No	124 (67.8)	533 (97.1)	657 (89.8)
Yes	59 (32.2)	16 (2.9)	75 (10.2)
*Receiving health interventions*			
Yes	83 (45.4)	414 (75.4)	497 (67.9)
No	100 (54.6)	135 (24.6)	235 (32.1)

^a^Time required to go to a water source from a house and return (round trip)

^b^Burning, composting, or dumping in a pit

^c^Sharing bed or cloth for prolonged time or prolonged, direct skin-to-skin contact history with a suspected or confirmed case in the 2 months prior to the survey

### Multivariable modeling through matched analysis

Multivariable conditional logistic regression detected that unmarried status (AmOR = 2.71; 95% CI 1.30–5.65); inability to read and write (AmOR = 5.10; 95% CI 1.81–14.36); large family (≥ 5 occupants) (AmOR = 6.67; 95% CI 2.83–15.73); long time (> 30 min) for water collection (AmOR = 2.27; 95% CI 1.08–4.76); low daily water consumption (< 20 L/C/D) (AmOR = 6.69; 95% CI 2.91–15.37); open disposal of solid wastes (AmOR = 5.60; 95% CI 2.53–12.40); and absence of health interventions (AmOR = 2.98; 95% CI 1.39–6.39) were the statistically significant predictors of scabies infestation. However, occupation, HH wealth index, water source type, sanitation facility type, liquid waste disposal, bathing frequency, cloth washing frequency, using soap for bathing, cloth changing frequency, history with a scabies case, and cloth mixing history with scabies case were not statistically associated with scabies infestation (Table 3).

**Table 3:** Predictors of scabies infestation in rural areas of *Aneded District*, northwest Ethiopia, March–May 2021

Variables	Crude mOR (95% CI) ^a^	*P*-value	Adjusted mOR (95% CI)^b^	*P*-value
*Marital status*				
Married	1		1	
Unmarried	2.78 (1.87–4.15)	*P* < 0.0001	2.71 (1.30–5.65)	*0.008*
*Educational level*				
Able to read & write	1		1	
Unable to read & write	5.62 (3.33–9.49)	*P* < 0.0001	5.10 (1.81–14.36)	*0.003*
*Occupation*				
Farmer & merchant	1		1	
Daily laborer	2.03 (1.04–3.96)	0.037	3.75 (0.82–17.11)	0.088
*Family size*				
1–4 individuals	1		1	
≥ 5 individuals	10.90 (6.73–17.65)	*P* < 0.0001	6.67 (2.83–15.73)	*P* < *0.0001*
*HH wealth index*				
Rich	1		1	
Poor	0.62 (0.38–1.01)	0.053	0.45 (0.17–1.19)	0.106
*Water source type*				
Public standpipe	1		1	
Unprotected spring & well	5.70 (3.63–8.96)	*P* < 0.0001	0.89 (0.37–2.11)	0.787
*Water collection time*				
1–30 min	1		1	
> 30 min	6.53 (4.38–9.73)	*P* < 0.0001	2.27 (1.08–4.76)	*0.031*
*Daily water consumption (average)*				
≥ 20 L/C/D	1		1	
< 20 L/C/D	8.72 (5.57–13.64)	*P* < 0.0001	6.69 (2.91–15.37)	*P* < *0.0001*
*Sanitation facility type*				
Pit latrine with cover slab	1		1	
Pit latrine without cover slab	4.25 (2.64–6.85)	*P* < 0.0001	1.36 (0.50–3.66)	0.544
*Solid waste disposal*				
Proper	1		1	
Open	4.67 (3.23–6.75)	*P* < 0.0001	5.60 (2.53–12.40)	*P* < *0.0001*
*Liquid waste disposal*				
Using soak pit	1		1	
Open discharging	0.91 (0.28–2.95)	0.879	NA	NA
*Bathing frequency*				
Weekly & below	1		1	
Every other week & above	2.40 (1.08–5.35)	0.032	2.76(0.28–26.88)	0.383
*Cloth washing frequency*				
Weekly & below	1		1	
Every other week & above	4.08 (1.66–10.07)	0.002	6.73(0.40–112.62)	0.185
*Using soap for bathing*				
Yes	1		1	
No	8.81 (4.32–17.94)	*P* < 0.0001	3.12 (0.66–14.64)	0.150
*Cloth changing frequency*				
Weekly & below	1		1	
Every other week & above	1.55 (0.69–3.47)	0.289	*NA*	*NA*
*History with a scabies case*				
No	1		1	
Yes	20.6 (10.62–39.95)	*P* < 0.0001	4.17 (0.98–17.70)	0.053
*Cloth mixing history with scabies case*				
No	1		1	
Yes	15.34 (8.05–29.25)	*P* < 0.0001	1.88 (0.40–8.71)	0.421
*Receiving health interventions*				
Yes	1		1	
No	5.14 (3.38–7.83)	*P* < 0.0001	2.98 (1.39–6.39)	*0.005*

*Crude mOR* crude matched odds ratio, *adjusted mOR* adjusted matched odds ratio; 1, reference category

^a^Denotes crude mOR using 95% CI in bivariable conditional logistic regression analysis

^b^Denotes adjusted mOR using 95% CI in multivariable conditional logistic regression analysis

NA, not applicable or not included in the adjusted analysis

The italics indicate statistically significant *P*-values in the adjusted analysis

## Discussion

In this study, being unmarried, illiterate, having a large family, long travel time for water collection, low daily water consumption, open dumping of solid wastes, and inaccessibility of scabies-specific interventions were statistically significant predictors of scabies infestations in rural areas of *Aneded* District, northwest Ethiopia.

Unmarried individuals and individuals in separated families had higher odds of scabies infestation than married individuals and those in married families. This finding is similar to a finding by Dagne and his colleagues [14]. This could be attributed to poor socioeconomic status in rural communities resulting from the inability to manage HH and individual activities, including personal hygiene and domestic hygiene, while living alone or in separated families. In addition, it is possible that this is due to the high prevalence of scabies among youths who are not normally at the age of marriage. Moreover, it is most likely linked with inadequate personal hygiene of separated families’ children who commonly employ at rich HHs to herd domestic animals. Several studies have reported a high attack rate of scabies among children and young adults [3, 4, 19, 39, 40], and this disease burden is attributed to poor personal hygiene of children in rural areas [14, 15, 41].

Individuals who could not read and write or individuals in illiterate families were at increased odds of scabies infestation. This corroborates the findings of other studies in Ethiopia [14, 17] and elsewhere in the world [9, 10, 15, 16, 42]. This might be because individuals who have no education are less informed about the benefits of good personal hygiene, dangers of living in overcrowded HHs, transmission of infectious diseases through sharing of clothes or bedding, or mixing up of undressed clothes. Therefore, they might be more vulnerable to being infested. The epidemiological disparity of scabies infestation due to poor personal hygiene, overcrowded living conditions, and sharing of fomites is well noted elsewhere [3, 8, 16].

A large family significantly increased the odds of scabies infestation in HHs compared to a small family. This finding is supported by studies undertaken in Ethiopia [3]. In Ethiopian rural communities, HH members have no separate rooms in a house that they use independently; hence, family size per se may be used as an indicator of overcrowding. By this definition, this finding could result from the overcrowded living of occupants in an HH. In addition, this might be due to the shortage of soap and detergents to maintain personal hygiene, sharing of beds, plinths or nightclothes in large family HHs. It is well documented that overcrowding can make individuals vulnerable to scabies [9, 16, 19]. However, a study conducted among students in Cameroonian boarding schools reported a statistically significant association of scabies infestation with small number of students per dormitory (≤ 10) [21]. The paradox of findings could come due to variation in study setting, study population, study design and habits of maintaining adequate personal hygiene among study populations. Moreover, the findings of a study in boarding schools might be confounded by unmeasured variables.

HHs that lasted a long time for water collection were at elevated odds of scabies infestation. Rural, hard to reach, sparsely populated and poor communities commonly do not have water sources for domestic purposes within a short walking distance. For this reason, this finding is highly likely linked to insufficient personal hygiene, which might be practiced due to the inaccessibility, shortage or unavailability of safe water. Several studies have reported a high occurrence of scabies in impoverished rural communities [4, 9, 10]. It is widely noted that scabies is a poverty-driven disease elsewhere in the world [42]. The profound impact of inadequate water supply on individual hygiene and domestic hygiene is evidenced by various studies [43].

In this study, low daily water consumption also made individuals prone to scabies infestation, which can be interpreted as having inadequate water supply in these HHs to perform their routine activities, including washing clothes and body mostly with soap and cleaning the domestic environment. It is well known that scabies is a water-washed disease that is mainly characterized by water quantity rather than water quality [4]. In relation to this, evidence is documented on a substantial reduction in scabies through the practice of frequent washing of the body and clothes with soap [8, 9, 12, 16]. Thus, the possible reason for this finding is insufficient individual hygiene and domestic hygiene, which might result from water scarcity.

Open dumping of solid wastes significantly exposed individuals to scabies infestation compared with HHs that manage solid wastes properly. Similar evidence was reported from several studies [8, 22–24]. A community-based study in resource-poor communities identified residence in urban slums as a significant predictor of scabies occurrence [44]. This finding may be explained by the large family, low family income, illiteracy, poor family hygiene, or poor housing conditions of such HHs, which predisposes them to be infested by scabies mites.

HHs that had not received mass therapy, health education, training, and ongoing surveillance had higher odds of scabies infestation, which was also documented in previous studies [20, 21]. An intervention study among young students in religious schools in Dhaka, Bangladesh ascertained that mass treatment, weekly health education, and daily monitoring of personal hygiene had significantly reduced the prevalence rate of scabies [12]. A prospective follow-up study in the Solomon Islands also evidenced the significant role of MDA and intensive active case finding in reducing the prevalence of scabies [45]. Other studies also confirmed the efficacy of MDA for the control of scabies [27, 46, 47]. This may be due to poor personal hygiene and sharing of clothes associated with the absence of scabies-specific interventions.

This study has certain limitations. This study did not apply definitive diagnoses, including dermoscopy and/or skin scrapings by microscopy to observe mites, their eggs and/or fecal pellets, and burrows in the epidermis. However, these diagnostic techniques have relatively low sensitivities and require technical skill. Recently, a blood test has also been used for the diagnosis of scabies mites despite having low specificity and sensitivity. Our diagnosis was entirely based on a clinical assessment independently conducted by two experienced public health professionals. The seasonality of scabies was not taken into account which may mask independent predictors in different seasons. Recall bias could have been present during the measurement of receiving scabies-specific interventions. Residual confounding was not adjusted, but the influence on the findings was minimal.

## Conclusions

Illiteracy, being unmarried, large family, long travel time for water collection, low daily water consumption, open dumping of solid wastes, and inaccessibility of scabies-specific interventions are independent predictors of scabies infestation. Special emphasis should be put in place for illiterate individuals, unmarried individuals or separated families, and large family HHs while designing and delivering interventions to effectively prevent and control scabies infestations. Adequate water accessibility and promotion of environmental sanitation through information, education and communication; and social behavioral change communication are needed to improve personal hygiene. Scabies-specific interventions should be given equally and optimally for all HHs in rural communities of Ethiopia by considering educational status for the delivery of scabies information. Integrated programs, including combination MDA are needed to control multiple NTDs economically.

## Data Availability

Data will be accessible from the corresponding author upon request.

## Abbreviations

CI: Confidence interval
DALYs: Disability-adjusted life-years
HH: Household
IQR: Interquartile range
L/C/D: Liters/capita/day
MDA: Mass drug administration
AmOR: Adjusted matched odds ratio
NTD: Neglected tropical disease
OR: Odds ratio
PCA: Principal components analysis
WaSH: Water supply, sanitation, and hygiene
WHO: World Health Organization
